# Optimizing a Behavioral Sleep Intervention for Gynecologic Cancer Survivors: Study Design and Protocol

**DOI:** 10.3389/fnins.2022.818718

**Published:** 2022-03-04

**Authors:** Rina S. Fox, Julia S. Gaumond, Phyllis C. Zee, Karen Kaiser, Edward J. Tanner, Sonia Ancoli-Israel, Juned Siddique, Frank J. Penedo, Lisa M. Wu, Kathryn J. Reid, Sairam Parthasarathy, Terry A. Badger, Christine Rini, Jason C. Ong

**Affiliations:** ^1^Division of Community and Systems Health Science, University of Arizona College of Nursing, Tucson, AZ, United States; ^2^University of Arizona Cancer Center, Tucson, AZ, United States; ^3^Department of Medical Social Sciences, Northwestern University Feinberg School of Medicine, Chicago, IL, United States; ^4^Department of Neurology, Northwestern University Feinberg School of Medicine, Chicago, IL, United States; ^5^Center for Circadian and Sleep Medicine, Northwestern University Feinberg School of Medicine, Chicago, IL, United States; ^6^Robert H. Lurie Comprehensive Cancer Center of Northwestern University, Chicago, IL, United States; ^7^Department of Obstetrics and Gynecology, Northwestern University Feinberg School of Medicine, Chicago, IL, United States; ^8^Department of Psychiatry, University of California, San Diego, San Diego, CA, United States; ^9^Department of Preventive Medicine, Northwestern University Feinberg School of Medicine, Chicago, IL, United States; ^10^Department of Medicine and Psychology and Sylvester Comprehensive Cancer Center, University of Miami, Miami, FL, United States; ^11^Aarhus Institute of Advanced Studies, Aarhus University, Aarhus, Denmark; ^12^Division of Pulmonary, Allergy, Critical Care, and Sleep Medicine, Department of Medicine, University of Arizona, Tucson, AZ, United States; ^13^University of Arizona Health Sciences – Center for Sleep and Circadian Science, University of Arizona, Tucson, AZ, United States; ^14^Nox Health, Suwanee, GA, United States

**Keywords:** sleep disturbance, gynecologic cancer, behavioral sleep intervention, optimization, cancer survivorship

## Abstract

Sleep difficulties, particularly symptoms of insomnia and circadian disruption, are among the primary complaints of gynecologic cancer survivors before, during, and after treatment. Moreover, difficulty sleeping has been linked to poorer health-related quality of life and elevated symptom burden in this population. Although leading behavioral sleep interventions have demonstrated efficacy among cancer survivors, up to 50% of survivors are non-adherent to these treatments, likely because these interventions require labor-intensive behavior and lifestyle changes. Therefore, there is a need for more effective and acceptable approaches to diminish sleep disturbance among cancer survivors. This manuscript describes the methodology of a two-part study guided by the Multiphase Optimization Strategy (MOST) framework to identify a streamlined behavioral sleep intervention for gynecologic cancer survivors. Three candidate intervention components previously shown to decrease sleep disturbance will be evaluated, including sleep restriction, stimulus control, and systematic bright light exposure. Participants will be adult women with a history of non-metastatic gynecologic cancer who have completed primary treatment and who report current poor sleep quality. Fifteen participants will be recruited for Part 1 of the study, which will utilize qualitative methods to identify barriers to and facilitators of intervention adherence. Results will inform changes to the delivery of the candidate intervention components to promote adherence in Part 2, where 80 participants will be recruited and randomized to one of eight conditions reflecting every possible combination of the three candidate intervention components in a full factorial design. Participants will complete assessments at baseline, post-intervention, and 3-months post-intervention. Part 2 results will identify the combination of candidate intervention components that yields the most efficacious yet efficient 6-week intervention for diminishing sleep disturbance. This is the first known study to apply the MOST framework to optimize a behavioral sleep intervention and will yield a resource-efficient treatment to diminish sleep disturbance, improve health-related quality of life, and decrease symptom burden among gynecologic cancer survivors. ClinicalTrials.gov Identifier: NCT05044975.

## Introduction

### Sleep Disturbance in Cancer Survivorship

Sleep difficulties, particularly symptoms of insomnia and circadian disruption, are common among gynecologic cancer survivors (Westin et al., 2016; Campbell et al., 2019; Palagini et al., 2021), with recent research demonstrating an estimated prevalence greater than 80% (Pozzar et al., 2021). Sleep disturbance has also been associated with poorer health-related quality of life (HRQOL) and higher symptom burden in this population (Ross et al., 2020). Difficulty sleeping is often treated with Cognitive Behavioral Therapy for Insomnia (CBT-I), the gold-standard treatment for insomnia, including among those with comorbid conditions (Schutte-Rodin et al., 2008; Qaseem et al., 2016; van Straten et al., 2017). CBT-I is a multicomponent behavioral intervention consisting of cognitive restructuring, sleep restriction, stimulus control, sleep hygiene education, and relaxation therapy (Morin and Benca, 2012). Sleep restriction and stimulus control are generally recognized as the core components driving treatment effects (Morin et al., 2006). A recent meta-analysis evaluating the efficacy of CBT-I specifically in cancer survivors found that the intervention improved multiple sleep outcomes, including sleep efficiency, sleep onset latency, wake after sleep onset, and insomnia symptom severity (Johnson et al., 2016). Another recent meta-analysis supported the efficacy of CBT-I among cancer survivors regardless of whether the intervention was delivered in-person or remotely (Ma et al., 2021). However, although CBT-I can be highly efficacious, up to 50% of cancer survivors do not adhere to the treatment as it is currently packaged, and thus do not benefit maximally from it (Matthews et al., 2012, 2013; McChargue et al., 2012; Garland et al., 2014). This may be because the intervention requires patients to make multiple, simultaneous, and often intrusive behavioral and lifestyle changes (Agnew et al., 2021). A less burdensome alternative is systematic bright light exposure, which has recently been explored as another strategy for improving sleep disturbance in cancer survivors (Wu et al., 2018, 2021; Fox et al., 2021). Results suggest systematic bright light exposure in the morning may have a beneficial impact on sleep disturbance among cancer survivors. However, effects are generally not as strong as they are for CBT-I, with research thus far limited to small pilot studies where the mechanisms underlying the effects of bright light have not been well explored.

A leading theory of sleep-wake regulation is the two-process model (Borbely et al., 2016), which posits that sleep timing and intensity depend on the interaction of a homeostatic process and a circadian process, which function independently. According to this model, the homeostatic process reflects the body’s attempts to maintain balance, in that more time spent awake leads to increased pressure to fall asleep (Deboer, 2018). The circadian process, a pacemaker managed by the suprachiasmatic nucleus of the hypothalamus, determines one’s daily rhythm for sleep and wake, leading to increased sleepiness at certain times of the day and increased alertness at others (Deboer, 2018). Thus, sleep-wake cycles and circadian rhythms separately influence sleep (Borbely et al., 2016). Sleep restriction and stimulus control, the two core components of CBT-I, both target the homeostatic process. Conversely, systematic bright light exposure targets the circadian process. Therefore, combining sleep restriction, stimulus control, and bright light may have additive or even interactive effects. One known study is currently exploring this hypothesis (Bean et al., 2020); however, it is doing so exclusively among patients undergoing chemotherapy for breast cancer. Thus, the applicability of results to cancer survivors who have completed primary treatment will be unclear. Additionally, this known study is adding systematic bright light exposure to CBT-I as a packaged intervention, thus increasing the burden associated with this already challenging treatment. To maximize benefit from the available evidence-based treatments, studies are needed that can determine what combination of behavioral sleep intervention components yields the most effective intervention while also maximizing adherence to optimize impact.

### Intervention Optimization

Multicomponent behavioral interventions are traditionally developed and evaluated as pre-bundled treatment packages and assessed *via* randomized controlled trial (RCT; Guastaferro and Collins, 2019). This is an effective and important scientific approach to identify how an intervention performs relative to a control or comparison. However, this approach is unable to identify which components of the intervention, if any, could be changed or removed to increase efficiency and decrease burden. That is to say, the effects of the individual components of the intervention, both independently and interactively, remain unknown. To address this challenge, Collins and colleagues developed the Multiphase Optimization Strategy (MOST) framework (Collins et al., 2005; Collins, 2018). MOST is grounded in engineering theory and involves three phases. Like traditional RCT-based intervention science, the MOST framework involves a Preparation phase that includes developing a conceptual model, selecting candidate intervention components based on theory and evidence, and conducting pilot testing. It also includes an Evaluation phase, in which components are bundled into a final intervention package that is then evaluated, typically *via* RCT.

What sets MOST apart is the inclusion of an Optimization phase, which occurs between the Preparation and Evaluation phases, in which candidate intervention components are evaluated using randomized experimentation to determine the impact of components individually and in various combinations. This enables identification of which components make valuable contributions to the overall program effects and what combination of components yields the greatest impact. The result is an intervention that has been optimized according to specific criteria (e.g., effectiveness, efficiency, cost) and that can achieve desired results with fewer resources and less participant burden. Of note, factorial designs are frequently used in the Optimization phase because they provide an economical framework for testing multiple components in a single study.

### The Present Study

This paper describes the procedures and methodology of a two-part study guided by the MOST framework. The first part, which is consistent with the Preparation phase of the framework, will employ a qualitative approach to identify barriers to and facilitators of adherence to sleep restriction, stimulus control, and systematic bright light exposure, three evidence-based candidate intervention components previously shown to decrease sleep disturbance among cancer survivors. The second part, which is consistent with the Optimization phase of the framework, will employ a factorial design to identify the best combination of these three components, enhanced to promote adherence per the results of Part 1, to yield a resource-efficient behavioral sleep intervention with exclusively active components. Feasibility, acceptability, and adherence will also be examined, and the potential mediating roles of sleep disturbance and circadian markers on intervention effects will be explored. This work will yield an optimized behavioral sleep intervention to diminish sleep disturbance, improve HRQOL, and reduce symptom burden among gynecologic cancer survivors.

## Materials and Methods

### Participants and Recruitment

Across both parts of the study, participants will be survivors of a non-metastatic gynecologic cancer who have completed primary treatment, are between the ages of 18 and 74, and report poor sleep quality. Part 1 of the study will include 15 participants and Part 2 will include 80, for a total sample size of 95 participants to be enrolled over 5 years. Participants will be recruited from the Robert H. Lurie Comprehensive Cancer Center of Northwestern University and the University of Arizona Cancer Center, two comprehensive cancer centers located in Chicago, IL and Tucson, AZ, United States, respectively. All potential participants will provide verbal consent to complete screening for study eligibility. Those who are eligible and subsequently enroll will provide written informed consent prior to engaging in any study activities.

### Eligibility

Potential participants will be identified through a combination of physician referral and electronic medical record screening. Study eligibility will be confirmed by participant self-report *via* a telephone screening interview. To be eligible for the study, participants must endorse or demonstrate: (1) a history of a non-metastatic ovarian, uterine, vaginal, vulvar, or cervical cancer; (2) English language proficiency; (3) age 18–74 years; (4) typical sleep onset between 9:00pm and 3:00am; (5) poor sleep quality [i.e., score > 5 on the Pittsburgh Sleep Quality Index [PSQI] (Buysse et al., 1989)]; and (6) reliable telephone and internet access.

Participants who meet any of the following criteria will be excluded: (1) diagnosis of a second primary cancer other than a non-melanoma skin cancer; (2) diagnosis of significant neurological, physiological or psychological dysfunction that could impact study participation [e.g., active psychosis, glaucoma, HIV, epilepsy, current substance abuse, active suicidality]; (3) diagnosis of sleep apnea, restless legs syndrome, periodic limb movement disorder, or narcolepsy per self-report and medical record review; (4) completion of primary anti-cancer treatment (e.g., chemotherapy, radiation therapy) <30 days prior to participation or surgical intervention <60 days prior to participation; (5) significant mental or physical decline (i.e., ≥2 mistakes on the six-item version of the Mini Mental Status Exam [MMSE] (Callahan et al., 2002) or Eastern Cooperative Oncology Group [ECOG] (Oken et al., 1982) performance status score > 1); (6) shift work; and (7) plans to travel across meridians during participation. Exclusion criteria were selected due to their known impact on study outcomes or because of potential interference with study participation.

### Candidate Intervention Components and Intervention Delivery

The three candidate intervention components to be evaluated were selected according to the two-process model of sleep-wake regulation (Borbely et al., 2016). To further minimize burden, only the sleep restriction and stimulus control components of CBT-I will be evaluated while sleep hygiene education, cognitive restructuring, and relaxation therapy will not, as sleep restriction and stimulus control have been identified as primary mechanisms of CBT-I efficacy among adherent patients (Morin et al., 2006).

#### Sleep Restriction

Sleep restriction involves limiting one’s opportunity for sleep to the amount of time reported sleeping on an average night plus 30 min. For this study, wake time will be determined by asking participants to identify the earliest time they need to awaken on any given day based on their individual lifestyles. The prescribed bedtime will then be identified by working backward from this wake time. The initial sleep opportunity window will be informed by 7 days of sleep diaries completed immediately prior to beginning the intervention. This window will then be adjusted weekly based on participants’ sleep efficiency (i.e., ratio of time asleep to time in bed at night), calculated from weekly sleep diaries, as is the gold standard recommendation (Spielman et al., 1987).

#### Stimulus Control

Based on classical and operant conditioning, stimulus control aims to disassociate non-sleep behaviors from the bedroom and reinforce the association of the bedroom with sleep-related stimuli (e.g., bed). Instructions include not going to bed until sleepy, getting out of bed if unable to fall asleep or fall back to sleep within approximately 20 min, avoiding behaviors other than sleep and sexual activity in the bedroom, waking up at the same time each day, and avoiding daytime naps (Bootzin et al., 1991).

#### Systematic Bright Light Exposure

Bright light will be systematically delivered by wearable Re-Timer™ glasses. The Re-Timer™ glasses are a small (7.9″×5.5″×2.2″), lightweight (2.64 oz.) device that delivers green-blue light from below to replicate the natural pathway of light to the eyes. Participants will be instructed to wear the glasses on the highest setting for 30 min every morning during the intervention period, initiating this time within 30 min of awakening and completing light exposure prior to noon. Re-Timer™ glasses can be worn over traditional eyeglasses as long as the eyeglasses do not have photochromatic or tinted lenses.

#### Intervention Delivery

Prior to the initiation of the intervention period, research staff will provide an intervention orientation to participants. This orientation, which will last approximately 30–60 min, will be completed either in-person or over video conference, and will include general sleep education as well as an explanation of the intervention components the participant will receive and the rationale behind them. After this orientation, participants will implement their assigned intervention components at home daily for 6 weeks.

The candidate intervention components will be administered *via* weekly emails. All participants will monitor their sleep by completing a daily sleep diary for the duration of the 6-week intervention period. All participants will also receive a weekly email from the study team with a reminder to complete and return the sleep diary. Sleep diaries will be returned electronically. For participants receiving stimulus control, the weekly email will also contain a reminder of the stimulus control instructions. For participants receiving systematic bright light exposure, the weekly email will also contain a reminder of how to use the Re-Timer™ glasses. For participants receiving sleep restriction, the weekly email will also contain a reminder of the sleep restriction instructions and the prescribed sleep schedule for the coming week, which will be determined by a licensed clinical psychologist with specialized training in behavioral sleep intervention (RSF) based on the prior week’s sleep diary. Participants receiving more than one candidate intervention component will receive a single weekly email containing the information relevant to all components being delivered. The study team will be available to participants throughout the intervention period to answer questions, troubleshoot, and provide support as needed.

### Study Design

This two-part study is guided by the MOST framework. Participants in Part 1 will not be randomized, but rather will receive all three candidate intervention components simultaneously. It is well established that greater adherence predicts behavioral sleep intervention efficacy; however, knowledge of how best to increase adherence is limited (Matthews et al., 2012, 2013; Agnew et al., 2021). Therefore, upon completion of the intervention in Part 1, individual semi-structured interviews will be conducted with participants and analyzed to identify barriers to and facilitators of adherence to the three candidate intervention components. Results will inform the design and delivery of the components in Part 2 to enhance adherence. Subsequently, in Part 2, a full factorial experiment will be completed that involves eight experimental conditions (Table 1). It is important to note that this study is not an eight-arm RCT and the purpose is not to compare outcomes across each of the eight conditions directly. Rather, each main effect and interaction estimate will use data from participants in all of the experimental conditions. For example, the main effect of sleep restriction will be estimated by comparing the mean for the 40 participants in conditions 1 through 4 to the mean for the 40 participants in conditions 5 through 8 in Table 1. A detailed explanation of how factorial study designs maintain power has been previously published (Collins et al., 2009).

**TABLE 1:** Experimental conditions.

Experimental condition	Sleep restriction	Stimulus control	Bright light
1	Yes	Yes	Yes
2	Yes	Yes	No
3	Yes	No	Yes
4	Yes	No	No
5	No	Yes	Yes
6	No	Yes	No
7	No	No	Yes
8	No	No	No

### Hypotheses

We hypothesize that gynecologic cancer survivors will be able to engage with each of the three candidate intervention components and that each component will be deemed acceptable. We further hypothesize that the optimal combination of intervention components will yield stronger effects for diminishing sleep disturbance, improving HRQOL, and reducing symptom burden than each individual component.

## Data Collection and Measures

### Data Collection

Data to be collected include sociodemographic information, medical information, patient-reported outcome measures, qualitative patient feedback, urine samples, and objective sleep information recorded by actigraphy. Throughout the study, the REDCap (Harris et al., 2009) data collection platform will be used to capture and store participant data. This platform is optimized for secure research data collection and storage and allows for HIPPA-compliant export and analysis. Data will be entered into REDCap either directly by participants or by the research team. All study assessments will be completed in-person or remotely *via* videoconference.

Across both Part 1 and Part 2 of the study, the screening questionnaire will be administered by telephone to determine eligibility. Eligible and interested participants will then provide informed consent before completing a baseline assessment where they will provide sociodemographic and medical information and complete questionnaires related to their sleep, HRQOL, and symptom burden. Participants will also collect urine the night before this baseline assessment, wear a wrist actigraph, and complete sleep diaries for the 7 days leading up to the assessment. Immediately after completing the 6-week intervention period, participants will complete a follow-up assessment that mirrors the baseline plus an additional measure of acceptability. In addition to the baseline and post-intervention assessments, participants in Part 1 will complete a semi-structured interview and accompanying survey within 2 weeks of completing the intervention to provide information about barriers to and facilitators of protocol adherence. In contrast, participants in Part 2 will complete an additional follow-up assessment 3 months after completing the intervention that mirrors the baseline and post-intervention assessments to measure longitudinal intervention effects.

### Measures

#### Feasibility and Acceptability

In both Part 1 and Part 2, study feasibility will be assessed according to established procedures (Bowen et al., 2009), including documenting the number of eligible participants, participant willingness to be enrolled, attrition, reported reasons for withdrawing, and self-reported protocol adherence.

Acceptability of the candidate intervention components will be assessed with items from the Functional Assessment of Chronic Illness Therapy – Treatment Satisfaction – General scale (FACIT-TS-G) (Peipert et al., 2014), which will be administered at the post-intervention assessment only. The FACIT-TS-G includes eight items that utilize Likert-type response scales, were developed as stand-alone single items, and are scored individually. Higher item scores indicate greater acceptability.

#### Covariates

##### Sociodemographic and Medical Information

Sociodemographic and medical information will be gathered through participant self-report and medical chart review at screening, baseline, post-intervention, and 3-month follow-up. Sociodemographic data will include age, education, income, racial background, ethnicity, employment status, and relationship status. Medical data will include information about exclusionary and comorbid medical conditions, prescription medication use, and cancer-related information (e.g., time since diagnosis, stage at diagnosis, treatment history). Participants will also complete the Self-Administered Comorbidity Questionnaire (Sangha et al., 2003).

##### Sleep Apnea Risk

The Berlin questionnaire (Netzer et al., 1999) will assess risk for sleep apnea at baseline. This screener consists of nine items reflecting known clinical features of sleep apnea and yields scores in three categories related to the risk of having sleep apnea. Participants are classified as High Risk or Low Risk based on their responses to individual items and their overall scores in the symptom categories.

##### Pain

An 11-item numerical rating scale, ranging from 0 (*No pain*) to 10 (*Worst imaginable pain*), will evaluate pain intensity (Ferreira-Valente et al., 2011) at baseline, post-intervention, and 3-month follow-up. The numerical rating scale is one of the most commonly used measures of pain intensity and has been shown to be responsive to pain (Ferreira-Valente et al., 2011).

##### Treatment Expectancy

Following the baseline assessment and orientation session and prior to the start of the 6-week intervention period, participants will complete the Credibility/Expectancy Questionnaire (Devilly and Borkovec, 2000). This six-item measure was developed for clinical studies to measure treatment expectancy and rationale credibility. It yields two subscale scores reflecting affectively based expectancy and cognitively based credibility.

#### Primary Outcomes

##### Objectively Measured Sleep

The Actiwatch Spectrum Plus (Mini Mitter/Phillips/Respironics) will be used to objectively measure sleep/wake activity at baseline, post-intervention, and 3-month follow-up. In addition to sleep/wake activity, the Actiwatch Spectrum Plus records information about amount and duration of ambient white light luminance in units of lux. Participants will wear the device, which is similar in size to a watch, on the non-dominant wrist for 7 consecutive days immediately prior to each assessment. Actiwatch Spectrum Plus data will be scored and analyzed with Actiware Sleep v6.1.2 (Phillips-Respironics, Mini Mitter, Bend, OR, United States) to calculate sleep outcomes, such as total sleep time, percent sleep, total wake time, number and duration of nighttime awakenings, sleep mid-point, and number and duration of daytime naps (defined as a minimum of 10 consecutive min of inactivity). These data will also be used to calculate circadian activity rhythms (Marler et al., 2006) and the sleep regularity index (Phillips et al., 2017), which are indicators of circadian rhythm robustness. Participants will be instructed to press the event marker on the Actiwatch when initiating sleep effort at the start of the main sleep period, when discontinuing sleep effort upon final awakening, and when taking daytime naps. When wearing the Actiwatch, participants will also complete an actigraphy log in which they will record instances of and reasons for Actiwatch removal, which will be used to inform editing of the actigraphy data (Ancoli-Israel et al., 2015).

##### Subjectively Measured Sleep

###### Consensus Sleep Diary

Participants will complete the Consensus Sleep Diary (Carney et al., 2012) daily during the intervention period and whenever wearing the Actiwatch Spectrum Plus. The Consensus Sleep Diary includes questions about time to bed, time to sleep, number and duration of nighttime awakenings, time of final awakening, time out of bed, and overall sleep quality. Diary data will guide the weekly sleep opportunity window for those receiving sleep restriction. It will also be used in conjunction with the actigraphy log to facilitate actigraphy scoring, as described above (Ancoli-Israel et al., 2015).

###### Perceived Sleep Quality

The eight-item Patient Reported Outcomes Measurement Information System (PROMIS) Sleep Disturbance Short Form 8a (Yu et al., 2012) will be used to assess perceived sleep quality at baseline, post-intervention, and 3-month follow-up. Respondents rate the quality of their sleep over the prior 7 days on a five-point scale ranging from *Very poor* to *Very good* and rate the frequency with which they experienced different sleep symptoms on a five-point scale ranging from *Not at all* to *Very much*. Higher scores reflect worse sleep quality. All PROMIS assessments generate *T* scores that are standard scores with a mean of 50 and a standard deviation of 10 based on the reference population in which the measure was developed. For many PROMIS measures this is the general United States population; however, the Sleep Disturbance scale was developed with a population enriched for chronic illness. Thus, a score of 50 on this scale represents the average for individuals slightly more impaired than the general population.

###### Sleep-Related Daytime Impairment

The eight-item PROMIS Sleep-related Impairment Short Form 8a (Yu et al., 2012) will assess perceived impairment during wake associated with sleep problems at baseline, post-intervention, and 3-month follow-up. Respondents rate the frequency with which they experienced daytime sleep-related symptoms over the prior 7 days on a five-point scale ranging from *Not at all* to *Very much*. Higher scores indicate greater daytime impairment. The PROMIS Sleep-related Impairment scale was also developed with a population enriched for chronic illness, and thus scores can be interpreted similarly to the Sleep Disturbance scale.

#### Secondary Outcomes

##### Health-Related Quality of Life

The 27-item Functional Assessment of Cancer Therapy – General (FACT-G) (Webster et al., 2003) will measure HRQOL at baseline, post-intervention, and 3-month follow-up. Participants indicate the extent to which each item has applied to them over the past week using a five-point scale ranging from *Not at all* to *Very much*, with higher scores indicating better HRQOL. The measure yields four subscales reflecting physical, social, emotional, and functional well-being, which can be summed to yield a total score.

##### Symptom Burden

###### The Memorial Symptom Assessment Scale

The 32-item Memorial Symptom Assessment Scale (MSAS; Portenoy et al., 1994) will evaluate overall symptom burden at baseline, post-intervention, and 3-month follow-up. The MSAS measures the prevalence of, severity of, and distress associated with 32 physical and psychological symptoms over the course of the past week. Frequency of symptoms is rated on a four-point scale ranging from *Rarely* to *Almost constantly*, severity of symptoms is rated on a four-point scale ranging from *Mild* to *Very severe*, and symptom-related distress is measured on a five-point scale ranging from *Not at all* to *Very much*. The measure yields three sub-scales reflecting physical symptom burden, psychological symptom burden, and overall distress. A total score can also be computed, with higher scores indicating greater symptom burden.

##### Patient Reported Outcomes Measurement Information System-Cancer Computer Adaptive Tests

Symptoms of fatigue (Lai et al., 2011), anxiety (Pilkonis et al., 2011), and depression (Pilkonis et al., 2011, 2014) will be assessed at baseline, post-intervention, and 3-month follow-up with PROMIS-Cancer computer adaptive tests. The PROMIS-Cancer scales were derived from established PROMIS item banks and were refined to increase the cancer-relevance of the measures (Garcia et al., 2007). For all constructs, respondents rate their symptom experience over the past 7 days on a five-point scale ranging from *Never* to *Always.* Select items in the Fatigue item bank use a five-point scale ranging from *Not at all* to *Very much* or from *None* to *Very.* Across all item banks higher scores indicate greater symptom burden.

#### Urine

Urinary 6-sulfatoxymelatonin (aMT6s), the primary urinary metabolite of melatonin, will be measured at baseline, post-intervention, and 3-month follow-up as a marker of circadian rhythms. The night prior to each assessment participants will collect three urine samples: pre-bedtime, overnight including the first morning void, and approximately 3 h after the first morning void (Cook et al., 2000). For each sample, total volume will be recorded, aMT6s and creatinine will be determined, and the concentration of aMT6s will be calculated and expressed as μg/g Cr (Schernhammer and Hankinson, 2009).

#### Adherence

In Part 1 of the study, participants will provide information about protocol adherence in the semi-structured interview and accompanying survey. In both parts of the study, adherence to sleep restriction and stimulus control instructions will be gathered by self-report and from the Consensus Sleep Diaries that participants will complete daily during the 6-week intervention period. Re-Timer™ usage and reasons for non-adherence will be recorded on a daily Re-Timer™ log (Fox et al., 2021).

### Analytic Plan

Across both parts of the study, descriptive statistics will be used to characterize the sample.

#### Part 1: Qualitative Approach

For Part 1 of the study, two coders will review the semi-structured interview transcripts and independently generate a preliminary list of themes regarding barriers and facilitators of treatment adherence using a constant comparative approach (Glaser and Strauss, 1967). The coders will meet regularly to discuss and compare themes. Redundant and irrelevant themes will be removed, and a coding dictionary will be developed for the remaining analysis. Coders will use this dictionary to code all transcripts, and data saturation will be evaluated. Past work indicates that saturation often occurs within the first 12 interviews, and basic themes may be identifiable as early as the sixth interview (Guest et al., 2006). Therefore, 15 interviews will be conducted to increase the likelihood of reaching saturation. Results, consisting of the most prevalent and important themes and corresponding quotations from participants, will inform any needed modifications to the delivery of the candidate intervention components to enhance adherence in Part 2.

#### Part 2: Intervention Optimization

For Part 2, we will use benchmarks of 80% recruitment (i.e., 80% of eligible survivors) and 80% retention to demonstrate feasibility. We will use a benchmark of 60% protocol adherence for each candidate intervention component, based on prior studies of CBT-I and bright light in cancer survivors (Berger et al., 2003; Ancoli-Israel et al., 2012). Descriptive statistics will be applied to the FACIT-TS-G items to examine acceptability.

To identify the optimal combination of candidate intervention components, longitudinal mixed effects models will be fit to the data with primary and secondary outcomes evaluated in separate models. All study time points will be included in these models, and fixed effects will include time (a nominal variable with three levels, which will be entered as two indicator variables), each candidate intervention component, and two-way interactions among components. The effect of a component at post-intervention and 3-month follow-up will be assessed based on that component’s interaction with time. Candidate intervention components will be defined using effect coding (absence: −1, presence: +1) so effects remain uncorrelated (Kugler et al., 2012). Models will also adjust for hypothesized covariates as outlined above, as well as demographic and baseline clinical characteristics found to significantly differ across conditions. Further analyses will evaluate if the presence of multiple candidate intervention components has a greater effect on each outcome than single components. To do this, an additional variable will be created to indicate the number of components to which a participant was exposed (0, 1, 2, 3). This variable will be included as a fixed effect in these models, along with its interaction with time.

In addition to optimizing the intervention, we will also explore the potential mediating role of circadian markers (i.e., aMT6s, circadian activity rhythms, sleep regularity) on the effects of candidate intervention components. The potential mediating role of sleep disturbance on the relationships of intervention components to HRQOL and symptom burden will also be evaluated. Given the exploratory nature of this analysis, interpretation will focus on effect sizes rather than statistical significance.

Power analyses for Part 2 were conducted using the Factorial Power Plan Macro for SAS (Dziak et al., 2013) to detect an effect size ≥0.40 for main effects and two-way interactions with 80% power and two-sided alpha = 0.05. Analyses will be conducted using a maximum likelihood estimation approach, which provides valid inference in the presence of missing data under a missing at random assumption (Enders and Bandalos, 2001). Once all data are collected, they will be imported into SPSS (IBM, Armonk, NY, United States) (IBM Corp, 2020) for cleaning. Data will then be analyzed in SPSS (IBM Corp, 2020), and MPlus (Muthén & Muthén, Los Angeles, CA, United States) (Muthén and Muthén, 1998-2017).

## Discussion

The goal of this study is to optimize a behavioral intervention to decrease sleep disturbance, improve HRQOL, and diminish symptom burden among survivors of gynecologic cancers. While efficacious when adhered to, current leading behavioral sleep intervention packages are burdensome and often have low adherence among oncologic samples. Part 1 of the study will address this concern by utilizing qualitative methods to identify barriers to and facilitators of intervention adherence, with results informing subsequent treatment delivery to maximize participant engagement. Additionally, delivering bundled treatment packages without assessing their individual components can lead to unnecessary burden and inefficient use of resources, because inactive components may be included in these treatments. By leveraging the MOST framework, Part 2 of this study will efficiently test the efficacy of three candidate intervention components independently and in combination to identify a resource-efficient, maximally effective, evidence-based behavioral sleep intervention for gynecologic cancer survivors that consists of exclusively active components. Feasibility, acceptability, and adherence will also be explored.

The primary limitation of this study is that intervention components that were not examined could be important to sleep disturbance, HRQOL, and/or symptom burden. However, we selected the components under evaluation because, taken together, they directly address both components of the two-process model of sleep-wake regulation (Borbely et al., 2016), emphasize the most well-supported components of CBT-I, and include a lower-burden systematic bright light exposure component that has shown promising efficacy and acceptability in past studies. Nonetheless, future research may benefit from examining other candidate intervention components. Additionally, it is possible that participants with non-insomnia sleep disorders may be recruited, as diagnostic sleep interviews are not being conducted as part of screening. However, to minimize the likelihood of this occurring we will assess past diagnosis of a sleep disorder by both self-report and medical record review.

To our knowledge, this is the first study that will apply the MOST framework to develop an efficient, minimally burdensome behavioral sleep intervention. Select prior studies have attempted to disentangle multicomponent sleep interventions like CBT-I using a comparative treatment design, in which participants receive an intervention component in isolation, a combination of components, or a control (Epstein et al., 2012; Harvey et al., 2014). However, unlike the present study, this approach has not supported exploration of interactions among components. The present study will use optimization strategies so the final treatment includes only active components, and intervention delivery will be adapted to promote treatment adherence. Given the notable impact and implications of sleep disturbance among gynecologic cancer survivors, there is a great need to identify non-pharmacological treatments to address symptoms with as little burden as possible. If the components ultimately included in the optimized intervention prove to be feasible and acceptable, this work will yield a resource-efficient behavioral intervention that is primed for evaluation in a subsequent, large, fully powered RCT. This in turn could identify an effective treatment package to diminish the highly prevalent and understudied problem of sleep disturbance among survivors of gynecologic cancers.

## Data Availability Statement

The original contributions presented in the study are included in the article/supplementary material, further inquiries can be directed to the corresponding author.

## Author Contributions

RF, PZ, KK, ET, SA-I, JS, FP, LW, KR, CR, and JO contributed to the conception and design of the study. RF wrote the first draft of the manuscript. JG wrote the sections of the manuscript. All authors contributed to manuscript revision, and read and approved the submitted version.

## Conflict of Interest

SP is a consultant for Jazz Pharmaceuticals, Inc., receives royalty from UpToDate, Inc., and has a patent that was licensed by SaiOx, Inc. (US20160213879A1). SP reports receiving grants to institution from the following entities: Sergey Brin Family Foundation (Verily Life Sciences, Inc.), Philips-Respironics, Inc., WHOOP, Inc., Sommetrics, Inc., and Regeneron, Inc. These conflicts are unrelated to this manuscript. The remaining authors declare that the research was conducted in the absence of any commercial or financial relationships that could be construed as a potential conflict of interest.

## Publisher’s Note

All claims expressed in this article are solely those of the authors and do not necessarily represent those of their affiliated organizations, or those of the publisher, the editors and the reviewers. Any product that may be evaluated in this article, or claim that may be made by its manufacturer, is not guaranteed or endorsed by the publisher.
